# SPODOBASE : an EST database for the lepidopteran crop pest *Spodoptera*

**DOI:** 10.1186/1471-2105-7-322

**Published:** 2006-06-23

**Authors:** Vincent Nègre, Thierry Hôtelier, Anne-Nathalie Volkoff, Sylvie Gimenez, François Cousserans, Kazuei Mita, Xavier Sabau, Janick Rocher, Miguel López-Ferber, Emmanuelle d'Alençon, Pascaline Audant, Cécile Sabourault, Vincent Bidegainberry, Frédérique Hilliou, Philippe Fournier

**Affiliations:** 1 Unité Informatique de Centre, INRA-AgroM, 2 place Viala, 34060 Montpellier Cedex 2, France; 2 Unité Biologie Intégrative et Virologie des Insectes, UMR1231, Université UMII, Bât. 24, cc101, place Eugène Bataillon, 34095 Montpellier Cedex 5, France; 3 Unité Résistance des Organismes aux Stress Environnementaux, UMR1112, INRA, 400 route des Chappes, BP167, 06903 Sophia-Antipolis Cedex, France; 4 Insect Genome Laboratory, National Institute of Agrobiological Sciences, 2-1-2 Kannondai, Tsukuba, Ibaraki 305-8602, Japan; 5 Unité Polymorphisme d'Intérêt Agronomique, Dép. AMIS, CIRAD, TA40/03, avenue d'Agropolis, 34398 Montpellier Cedex 5, France; 6 EMI 0229 INSERM, CRLC Val d'Aurelle, 34298 Montpellier Cedex 5, France; 7 Ecole des Mines, Départ. LGEI, 6 av. Clavières, 30319 Alès Cedex, France

## Abstract

**Background:**

The Lepidoptera *Spodoptera frugiperda *is a pest which causes widespread economic damage on a variety of crop plants. It is also well known through its famous Sf9 cell line which is used for numerous heterologous protein productions. Species of the *Spodoptera *genus are used as model for pesticide resistance and to study virus host interactions. A genomic approach is now a critical step for further new developments in biology and pathology of these insects, and the results of ESTs sequencing efforts need to be structured into databases providing an integrated set of tools and informations.

**Description:**

The ESTs from five independent cDNA libraries, prepared from three different *S. frugiperda *tissues (hemocytes, midgut and fat body) and from the Sf9 cell line, are deposited in the database. These tissues were chosen because of their importance in biological processes such as immune response, development and plant/insect interaction. So far, the SPODOBASE contains 29,325 ESTs, which are cleaned and clustered into non-redundant sets (2294 clusters and 6103 singletons). The SPODOBASE is constructed in such a way that other ESTs from *S. frugiperda *or other species may be added. User can retrieve information using text searches, pre-formatted queries, query assistant or blast searches. Annotation is provided against NCBI, UNIPROT or *Bombyx mori *ESTs databases, and with GO-Slim vocabulary.

**Conclusion:**

The SPODOBASE database provides integrated access to expressed sequence tags (EST) from the lepidopteran insect *Spodoptera frugiperda*. It is a publicly available structured database with insect pest sequences which will allow identification of a number of genes and comprehensive cloning of gene families of interest for scientific community. SPODOBASE is available from URL:

## Background

Lepidoptera represent a diverse and important group of agricultural insect pests that cause widespread economic damage on food and fiber crop plants, fruit trees, forests, and stored grains. They are also important indicators of ecosystem diversity and health. Moreover, lepidopteran insects display experimental advantages such as their large body size, accessible genetics, and extreme diversity. They show a large spectrum of interactions with plants and with numerous parasites or pathogens. Among Lepidoptera, the genus *Spodoptera *is largely studied due to its wide geographical distribution area. Indeed *Spodoptera *species are scattered over all continents. Presence *of S. frugiperda *in the American continent and in the Caribbean area has been studied in detail [1,2]. *S. frugiperda *larvae cause severe damage on many cultivated crops including corn, rice and maize. *S. littoralis *is reported to cause damages in Mediterranean and African subtropical regions as well as in China whereas *S. litura *is found in India, Indonesia and Australia. In addition to being important agricultural pests these noctuids are biological models studied for several purposes. For example,*S. frugiperda *is well known through its famous Sf21 cell line and its Sf9 subclone [3] which is used for numerous heterologous protein productions. *S. frugiperda *is also used to study pesticide resistance [4,5] and baculovirus host interaction [6], whereas *S. littoralis *is a model species to study pheromone regulations [7-9] or densovirus pathogenicity [10].

The development of new methods of insect pest management is an important challenge for world economy and health and it will be facilitated by a better knowledge of lepidopteran crop pest genomics. Indeed, genome information provides powerful tools for understanding biological mechanisms and functions and is essential for biology, medical science, and agriculture.

Recent years have shown a tremendous development of genome projects of various species, in particular for insects. Among model organisms, genome sequences have been completed in *Drosophila melanogaster *[11], the malaria mosquito, *Anopheles gambiae *[12], the honeybee *Apis mellifera *[13] and the silkworm *Bombyx mori *[14,15]. In the year 2002 an International Lepidoptera Genome Consortium was created, which gathers the cooperative efforts of various laboratories in the world on genomic and transcriptomic studies on insects of scientific and economic importance [16]. The project is organized in a "Bombyx – Plus" scheme, where *Bombyx mori *represents the core node of the knowledge both in terms of genetics, physiology, and EST sequencing [17]. Around this model, the genomic study of a variety of pests of agronomical importance has been encouraged, as functional genomics analysis were still limited by the lack of relevant genome databases for gene identification. Several EST sequencing projects have already begun, but the results of only a few are available, as for example on *Choristoneura fumiferana *[18], *Helicoverpa armigera *[19], *Plutella xylostella *or *Manduca sexta *[20]. Some other butterflies are also investigated [21]. We have developed resources for *Spodoptera frugiperda*, for which we have created a genomic BAC library [22] and a set of Expressed Sequence Tags (ESTs) from the well known Sf9 cell line [23]. Other labs have also reported the development of ESTs collections (Rollie J. Clem, Kansas State University, pers. comm.).

Here we present the database, named SPODOBASE, which provides integrated access to expressed sequence tags (EST) from *S. frugiperda*. The SPODOBASE currently contains 29,325 sequences from various organs (Sf9 cell line, hemocytes, midgut and fat body tissues). The EST sequences were cleaned and clustered into non-redundant sets (2294 clusters and 6103 singletons). User can retrieve information using text searches, pre-formatted queries, query assistant or blast searches.

This database will enable future functional genomics studies of a variety of biological processes such as immunity, endocrinology, reproduction or behavior. Since several physiological processes have been shown to be conserved through evolution, their study in lepidopteran models will help to further elucidate the function of homologous genes and will provide complements to the model insects *Drosophila *and *Anopheles*. For example these two model insects lack the receptors for the largely used *Bacillus thuringiensis *toxin as well as for most of the chemical pesticides (acetyl cholinesterase type). One can predict that analysis of the lepidopteran crop pests will contribute to sustainable agriculture, protection of the environment and maintenance of biodiversity.

## Construction and content

### 1. Construction of cDNA libraries and sequencing

Four directional cDNA libraries were generated for *Spodoptera frugiperda *larvae. A Sf9 cell line library has been previously constructed and described [23]. To generate the new libraries, different tissues of last larval instars, circulating hemocytes, fat body and midgut, were collected directly in TRIZOL reagent (InVitroGen). Extracted total RNAs were reverse transcribed using the SMART cDNA library Construction Kit (Clontech) according to manufacturer instructions. The library was built in λ Triplex2. From the phages, excision and circularization of pTriplEx plasmid was heat-induced at *lox*P sites in order to generate a plasmid library to be sequenced. The clones were robot-picked from agarose plates (CIRAD platform, Montpellier) and stored in 20% glycerol LB medium in 96-wells plates. A total of 72, 126 and 191 plates were seeded for the hemocyte (H), fat body (F) and midgut (M) libraries, respectively.

The 37,344 bacterial clones were then spotted on high density Nylon membranes and hybridized with an oligonucleotide probe encompassing the multiple cloning site in order to detect empty plasmid clones. Hybridization was conducted at high stringency and allowed the elimination of around 30 % clones in the different libraries. After colony picking, a limited sequencing test on 1900 clones from the 3 libraries revealed that the percentage of clones without insert was around 9%, showing an effective but non total rearrangement.

A second hybridization was performed with a probe consisting of a mixture of 40 cDNAs, in order to detect clones corresponding to cDNAs that were abundantly represented within the previously analyzed Sf9 library. We were expecting to increase coverage and decrease the number of sequences corresponding to known housekeeping genes. This hybridization leaded to the elimination of 0.7%, 1.9 % and 4.4 % of the clones in F, M and H libraries, respectively. We observed (See A) that the abundance of these clones was significantly reduced by this procedure, as their percentage in the library decreased from 36 % in the initial Sf9 library to 11 % in the four tissues libraries. Elimination was not total, probably because the complex probe does not detect easily all of the 40 genes, but it was still useful to avoid useless sequencing.

To assess inserts size, DNA was extracted from 96, 48 and 48 clones from the H, F and M libraries, respectively using the Qiagen DNA extraction kit. Inserts were amplified by PCR using primers flanking the insert cloning sites and their size was controlled by agarose gel electrophoresis. We found an average size of 1.1, 1.0 and 0.9 kb for the *S. frugiperda *cDNAs from the H, F and M libraries, respectively.

The libraries were thus re-assorted in a total of 55 plates for the hemocyte library, 87 plates for the fat body library and 149 plates for the midgut library, stored in 5% glycerol 2YT medium, in duplicate. From those, 5184 (54 plates), 6048 (63 plates) and 5952 (62 plates) clones were subjected to sequencing for hemocyte, fat body and midgut libraries, respectively. The plasmid DNAs were extracted from overnight grown bacterial cultures using an automated plasmid isolation machine BIO ROBOT 8000 (Qiagen). The cDNAs were sequenced using ABI PRISM BigDye Terminator Cycle Sequencing Ready Reaction kits on an ABI PRISM 3700 DNA Analyzer (Applied Biosystems) in Insect Genome Laboratory of National Institute of Agrobiological Sciences (NIAS, Japan). All clones were sequenced from both 5' and 3' extremities using forward and reverse primers located in the pTriplex vector, in a region flanking the insert. We thus obtained a total of 10,368, 12,096 and 11,904 sequences for hemocytes, fat body and midgut respectively.

A second midgut cDNA library was made from pooled mRNAs extracted from midguts of 3rd instar larvae fed on artificial diet supplemented with various natural products and xenobiotics. This library generated a set of 2,688 sequences.

### 2. The SPODOBASE pipeline

**Figure 1:**
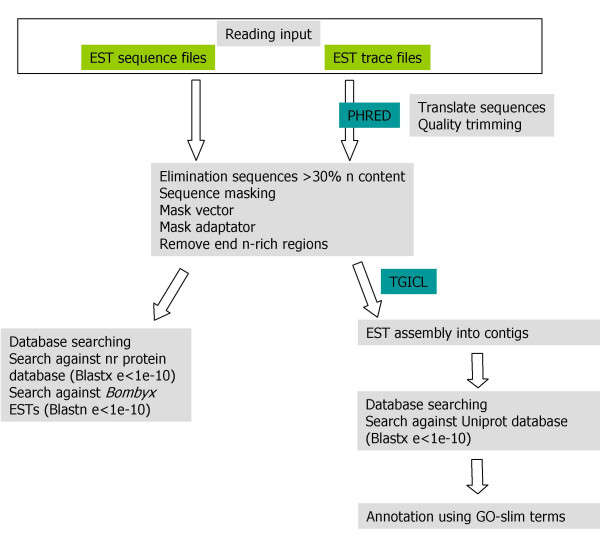
SPODOBASE EST pipeline flow chart.

Once the sequences established, they were analyzed and processed according to the flow chart depicted in Figure 1. The pipeline developed for EST analysis was divided into three steps: EST quality control, clustering and annotations.

#### 2-1 EST quality control

The sequences were given a unique ID consisting of a prefix including the species (Sf), 1 digit for the library number, the tissue origin (H, M, F or SF9L), 5 digits for clone number, 1 for sequencing direction and 1 for walking number. Sequences were then subjected to quality checking. Base calling step was performed using the Phred program [24,25]. Low quality bases (phred score < 10; this quite permissive score was chosen due to the low quality of some of the EST sequences) were masked and sequences with more than 30 % n-content were removed. The vector sequences were detected and removed. For this, we used BLASTN [26] with the following parameters (-q -5 -G 3 -E 3 -F "m D" -e 700 -Y 1.75e12). Due to their short length (less than 20 bp), the adaptor sequences were detected with an exact and more sensitive local alignment algorithm (Miller-Myers algorithm) and then eliminated. The regions of the sequences that contained more than 15 N's on a 20 bases window in the first/last quarters of the sequence were removed on both ends. The sequences with nucleotide stretches, indicators of sequences of bad quality, were also removed. Lastly, the cleaned sequences shorter than 100 bp were eliminated. After the cleaning process, we obtained a total of 23,503 sequences, representing 63% of the initial 37,056 EST sequences. With the 5822 EST already available from the Sf9L library, SPODOBASE contains a total of 29,325 ESTs, which are in majority 500–600 bp long. The distribution of EST sequences according to tissue origin is given in Table 1. Sequencing was conducted in both directions for all ESTs coming from tissues (Sf9 clones had only be 5' sequenced), but both sequences were not always retained after quality control, especially at the 3' end. The number of clones with available 5' only, 3' only or both sequences is given on Table 1, where one can see that 20163 clones have produced a readable sequence.

#### 2-2 EST clustering

**Figure 2:**
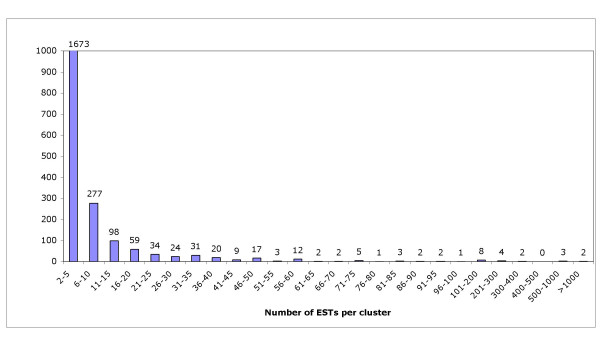
Distribution of the number of ESTs per cluster for the 2294 clusters. The number of EST is given for each class of abundance (2–5, 6–10, 11–15, etc).

All the 29,325 cleaned EST sequences were then subjected to clustering using the TIGR software TGI Clustering tool (TGICL) [27]. The clustering was performed by a modified version of NCBI's megablast. EST sequences were assigned to clusters based on identity: the clustering parameters were 98% minimum percent identity for overlaps, for a minimum overlap length of 40 nt and a maximum length of unmatched overhangs of 20 nt. The cluster names corresponded to the name of the first EST sequence assigned to the cluster. Thus, each cluster name will be maintained as additional ESTs are added to the database. After analysis, the 29,325 cleaned EST sequences were distributed among 2294 clusters and 6103 singletons. Most of the clusters (2141; 93%) contained 2 to 25 ESTs (Figure 2). In this step, 5' and 3' sequences are treated as independent data, so that sequences coming from the same clone may belong to two different clusters. This allows to control if a clone is not colinear to the genome (due to cloning artifact), or if the encoded gene contains similarities with two different genes. We then examined the clone origin of clusters and singletons and were able to deduce from these data a set of 5186 unigenes. As *Spodoptera *has a genome coding capacity (genome size 407 Mb, see ref. 22 comparable or slightly smaller than that of *Bombyx mori *(genome size 514 Mb for an estimated gene count of around 18,500; see refs. [14,15]], one can assume that the 5186 *Spodoptera *unigene collection described here represents at least 35 % of potential total gene number.

#### 2-3 EST assembling

Sequences from each cluster were assembled into consensus sequences called contigs using the CAP3 assembly program available in TGICL. By doing that, we found 97 clusters (4 %) that were separated in more than one contigs (Table 2) leading to a final number of 2436 contigs instead of the 2294 clusters described above. This discrepancy can be explained by small differences in the EST sequences probably due to transcript diversity (mutations, deletions). Note that sequences from a cluster containing only one sequence are called singletons.

#### 2-4 EST annotation

To identify similarities with known proteins, the sequences were searched using the BLASTX algorithm against a local non-redundant protein database (NR, NCBI, release 151.0, 1^st ^February 2006) with a cut-off E-value of 1e-10. A total of 18,736 (64 %) sequences were found to share significant similarity with a protein sequence deposited in the NCBI non-redundant database.

As genome data (including ESTs) of *Bombyx mori *are the most important among Lepidoptera, it represents a model organism within this order. We thus subjected the EST sequences to TBLASTX searches against 116,541 *B. mori *sequences deposited in the NCBI dbEST database with a cut-off E-value of 1e-10. A total of 21,185 (72%) sequences were found to share significant similarities with silkworm EST sequences.

Thus, 24 % (8141) of the *S. frugiperda *ESTs do not have a match in BLAST searches against neither NCBI nr nor *Bombyx mori *databases. To identify those that did not match because they may correspond to untranslated regions, a search for predicted coding regions was performed with the software ESTScan [28]. Indeed, from these 8141 sequences, we identified 3624 sequences (44.5 %) lacking predicted coding regions. Consequently, only 15 % of all sequences should be considered as new sequences. At this stage it should also be emphasized that *B. mori *ESTs database do not represent the total number of putative silkworm genes, thus the TBLASTX should be conducted against whole *B. mori *genome when it will be annotated. This observation may also be correlated with the phylogenetic distance which separates the two species. Indeed, although the monophyletic origin of Lepidoptera is well admitted [29], Bombycoidea and Noctuidea are two well distinct super families among this order, separated by probably more than 60 million years [30-32].

We also compared the 2436 contigs and the 6103 singletons to the Uniprot [33] protein database (release 6.0, September 2005) using the BLASTX program with a 1e-10 cut-off. We found 1178 contigs (48%) and 1809 singletons (30%) that showed a significant similarity with a Uniprot entry.

#### 2-5- GO assignment of the EST sequences in the SPODOBASE

To define the function of the contigs and singletons present in the SPODOBASE, we used the Gene Ontology (GO) controlled vocabulary [34], and more particularly GOSlim, a subset of GO terms, which provides a higher level of annotations and allows a more global view of the dataset. To this end, we searched for the GOSlim terms (provided by GOA [35] released on January 2006) associated with the 1178 contigs and 1809 singletons that showed a significant similarity with a Uniprot entry. These identifiers were further used to select the sequences to be printed on a *Spodoptera *DNA microarray (R. Feyereisen, pers. comm.).

#### 2-6- Software

The database is based on the AceDB database management system version [36], originally created for the worm *Caenorhabditis elegans*, and used by many databases: WormBase [37], crop-related databases available from the UK Crop Plant Bioinformatics Network WWW site [38], MagnaportheDB [39], ESTHER [40], ParaDB [41], TropGene [42], etc. This is an object-oriented system capable of storing and retrieving complex biological information. The Web server is an Apache Web server version running on Red Hat Linux version. The Web consultation interface is implemented with Perl/CGI scripts, using modules of the AcePerl Application Programming Interface (API) and the AceBrowser generic web interface [43]. The EST pipeline was created with Perl programming language and Bioperl libraries and used additional programs (PHRED for sequence quality control, BLAST for contaminant detection and annotation step, TGICL for clustering and assembling).

## Utility and discussion

### 1- User interface

For each sequence, series of information are available including the direction of sequencing, the existence of the other direction sequence, the relation to an existing cluster, the 10 best hits of BLASTX against NCBI and *Bombyx *EST database, and the library where the sequence was found. For each cluster, the software displays the distribution of sequences among the different tissue libraries, and gives the list of sequences belonging to the cluster; it offers the possibility to visualize their alignment and to download the FASTA file comprising all of them. The 10 best hits of BLASTX against Uniprot and G0 annotations are available for each contig and singleton. Users can query database in several ways. Information can be retrieved according to text search or using a query assistant.

#### 1-1- Classical AceDB queries

User can query database with AceDB data queries (Class, Text and AceDB queries). Class query allows the user to retrieve objects by class, with the possibility of restricting the search to names that match a pattern. Text query is a keyword-based search on all the data. AceDB query uses the Ace Query Language (AQL), which was created to formulate complex queries based on several criteria. In order to create an AQL request, the user must know the structure of the object model and learn a specific syntax. However some examples of classical questions written in AQL can be found at the AQLquery top page.

#### 1-2- Query assistant

To help the user for retrieval, we implemented the QueryBuilder tool [43]. This is a step-by-step graphic interface to formulate Ace queries. Five initial choices are proposed, concerning the clusters, the singletons, the libraries, the contigs or the sequences themselves. After this, the retrieval can be directed within a specific field and the chain of characters or numbers to be found are used in combination with the classical Boolean operators.

#### 1-3- BLAST search

Users can search for similarities between their own sequences using BLASTN, TBLASTN or TBLASTX searches against the whole set of *S. frugiperda *EST sequences.

### 2- Intended uses

The database provides an overview of *S. frugiperda *transcripts. One of the major interests of the SPODOBASE consists in the large number of sequences and the existence of 5 different tissues cDNA libraries. The database can be used, among other applications, for functional genomics (primer design for micro-array analysis), to identify the genes expressed predominantly in a given tissue, and to compare genes between different species. On the basis of extensive sequence-based analysis of relationships among noctuids, it has been recently shown [44] that *Spodoptera *is relatively close to a group of species called the "pest clade" and including Heliothinae and Noctuinae *s. l*. Actually SPODOBASE is constructed in such a way that it can welcome large numbers of additional sequences from other different tissues of *S. frugiperda*, as well as from other *Spodoptera *species. The implementation of *S. littoralis *ESTs is already programmed for a near future.

## Conclusion

The SPODOBASE represent a major contribution to the genomics of *Spodoptera frugiperda*. Together with BAC library, existence of various cell lines and expression systems, this makes of *S. frugiperda *of the most advanced models among agricultural pests in terms of genomic resources. SPODOBASE contains EST sequences that are cleaned, clusterized and annotated. These informations are available to serve insect research community, provide better understanding of the Lepidoptera physiology and identify new molecules targeted against Lepidoptera pests that could be used as safe biopesticides for sustainable agriculture.

## Availability and requirements

The database is publicly available at the following URL:. All sequences could be downloaded from SPODOBASE (see Download section). They have also been deposited in dbEST database (accession numbers for midgut library: DV075863 to DV080045 and DY786624 to DY7927772; fat body library: DY773453 to DY780623; hemocytes: DY773453 to DY780623; Sf9 cell line library: DY895775 to DY901596).

## Authors' contributions

VN, TH, MLF and FC constructed the database format and arranged the pipeline of algorithms that sequences should go through. VN and TH developed the user interface. SG, KM, XS, JR, EdA, PA, CS, VB and FH were involved in tissue mRNA isolation, library construction, replicating and storage of the clones, and ESTs sequencing. ANV analyzed the clusters, the GO classification, and wrote the paper initial draft. PF conceived and coordinated the study as responsible of the genomic *Spodoptera *program, and helped VN to draft the manuscript. All others agreed with the manuscript.

## Abbreviations

bp: base pairs

nt: nucleotide

EST: Expressed Sequence Tags

cDNA: copy DNA

GO: Gene Ontology

## Figures and Tables

**Table 1:** Tissue distribution of the number of clones having produced either 5' or 3' end sequencing or both, and subsequent EST numbers in SPODOBASE.

	clones with available			
	5' seq. only	3' seq. only	Both 5' & 3' seq.	Total nb of ESTs
Sf9L, Sf9 cell Line	5822	0	0	5822
Sf1F, Fat body	459	292	3210	7171
Sf1H, Hemocytes	491	357	2576	6000
Sf1M, Midgut	436	425	2644	6149
Sf2M, Midgut	2663	56	732	4183
				
Total per category	9871	1130	9162	29325

Total nb of clones		20163		

**Table 2:** Distribution of the number of contigs among the clusters. The final number of contigs is given.

	**nb of clusters**	**contigs/cluster**	**nb of contigs**
	2197	1	2197
	80	2	160
	7	3	21
	5	4	20
	2	6	12
	1	8	8
	1	9	9
	1	10	10

**total**	2294		2437

**Table A:** 

**clone/sequence**	**cluster**	**ID**	**Sf9L (clones)**	%	**tissue(clones)**	%	**total (clone)**
SF9LQ2237	SF9L00001	cytochrome b	142	2,4	145	1,0	287
SF9L01474	SF9L00002	ribosomal protein L8	27	0,5	21	0,1	48
SF9L02215	SF9L00003	ribosomal protein S23	21	0,4	6	0,0	27
SF9L01479	SF9L00004	cytochrome c oxydase subunit III	426	7,3	607	4,2	1033
SF9L02449	*SF9L00451*	ribosomal protein L14	30	0,5	8	0,1	38
SF9L01576	SF9L00008	ribosomal protein L23	178	3,1	20	0,1	198
SF9L01837	SF9L00009	ribosomal protein S11	42	0,7	16	0,1	58
SF9L01752	SF9L00014	ribosomal protein L35A	76	1,3	7	0,0	83
SF9L01773	SF9L00017	ribosomal protein L10A	29	0,5	6	0,0	35
SF9L01547	SF9L00018	ribosomal protein L24	32	0,5	10	0,1	42
SF9L03436	SF9L00024	ribosomal protein S14	26	0,4	17	0,1	43
SF9L01952	SF9L00027	cofilin	46	0,8	27	0,2	73
SF9L01583	SF9L00035	ribosomal protein L22	97	1,7	11	0,1	108
SF9L01582	SF9L00037	ribosomal protein L37A	49	0,8	6	0,0	55
SF9L03736	SF9L00045	ribosomal protein S8	38	0,7	9	0,1	47
SF9L02161	SF9L00047	NS	33	0,6	16	0,1	49
SF9L01896	SF9L00052	ribosomal protein L32	31	0,5	2	0,0	33
SF9L01859	SF9L00055	ribosomal protein S10	28	0,5	12	0,1	40
SF9L02679	SF9L00056	ribosomal protein L13A	25	0,4	15	0,1	40
SF9L01559	SF9L00084	cytochrome c oxydase subunit II	146	2,5	511	3,6	657
SF9L01151	SF9L00094	ribosomal protein L37	24	0,4	6	0,0	30
SF9L02183	SF9L00111	ribosomal protein S26	17	0,3	6	0,0	23
SF9L01846	SF9L00114	ribosomal protein S3A	40	0,7	15	0,1	55
SF9L01712	SF9L00118	ribosomal protein L39	22	0,4	11	0,1	33
SF9L02623	SF9L00122	ribosomal protein S25	19	0,3	4	0,0	23
SF9L02635	SF9L00143	ribosomal protein L28	49	0,8	1	0,0	50
SF9L01443	SF9L00144	ribosomal protein S17	40	0,7	8	0,1	48
SF9L01714	SF9L00174	ribosomal protein L31	36	0,6	5	0,0	41
SF9L02426	SF9L00176	ribosomal protein S13	32	0,5	4	0,0	36
SF9L03724	SF9L00197	ribosomal protein S4	42	0,7	12	0,1	54
SF9L01028	SF9L00240	ribosomal protein L27	23	0,4	5	0,0	28
SF9L01232	SF9L00323	ribosomal protein L40	35	0,6	15	0,1	50
SF9L01723	SF9L00334	ribosomal protein S12	32	0,5	3	0,0	35
SF9L01766	SF9L00336	ribosomal protein L36A	18	0,3	9	0,1	27
SF9L01498	SF9L00385	ribosomal protein S20	27	0,5	2	0,0	29
SF9L02368	SF9L00421	ribosomal protein S24	21	0,4	5	0,0	26
SF9L03196	SF9L00574	ribosomal protein L21	17	0,3	6	0,0	23
SF9L01548	SF9L00632	ribosomal protein L27A	62	1,1	10	0,1	72
SF9L02262	SF9L00705	ribosomal protein L13	25	0,4	12	0,1	37
SF9L02428	SF9L01994	ribosomal protein S3	23	0,4	7	0,0	30
		TOTAL	2126	S = 5822	1618	S = 14382	3744
		%	36,5		11,3		

Table A – Sf9L clones used to probe the tissue libraries. The clone (clone/sequence) and the cluster it belongs to, as well as the identity (ID) are given for each of the Sf9L clones used. Are also indicated the total number of clones found in the Sf9L and in the 3 tissues libraries (clones) and their percentage (%) compared to the total number of Sf9L clones and the 4 tissue libraries in SPODOBASE.
